# Efficacy and Safety of Albendazole in Hookworm-infected Preschool-aged Children, School-aged Children, and Adults in Côte d’Ivoire: A Phase 2 Randomized, Controlled Dose-finding Trial

**DOI:** 10.1093/cid/ciaa989

**Published:** 2020-07-15

**Authors:** Chandni Patel, Jean T Coulibaly, Daniela Hofmann, Yves N’Gbesso, Jan Hattendorf, Jennifer Keiser

**Affiliations:** 1 Department of Medical Parasitology and Infection Biology, Swiss Tropical and Public Health Institute, Basel, Switzerland; 2 University of Basel, Basel Switzerland; 3 Unité de Formation et de Recherche Biosciences, Université Félix Houphouët-Boigny, Abidjan, Côte d’Ivoire; 4 Centre Suisse de Recherches Scientifiques en Côte d’Ivoire, Abidjan, Côte d’Ivoire; 5 Centre de Santé Urbain d’Azaguié, Department de Agboville, Côte d’Ivoire; 6 Department of Epidemiology and Public Health, Swiss Tropical and Public Health Institute, Basel, Switzerland

**Keywords:** hookworm, Côte d’Ivoire, albendazole, soil-transmitted helminthiasis, drug safety

## Abstract

**Background:**

Infections with hookworms affect about half a billion people worldwide. Recommended therapy includes 400 mg of albendazole, which is moderately efficacious. Higher doses have been rarely assessed.

**Methods:**

A randomized, controlled dose-finding trial was conducted in Côte d’Ivoire with the aim of recruiting 120 preschool-aged children (PSAC), 200 school-aged children (SAC), and 200 adults. Eligible PSAC were randomized 1:1:1 to 200 mg, 400 mg, or 600 mg of albendazole; the other age groups were randomized 1:1:1:1:1 to placebo or 200 mg, 400 mg, 600 mg, or 800 mg. The primary outcome was cure rates (CRs) assessed 14–21 days post-treatment by quadruplicate Kato-Katz thick smears. Hyperbolic *E*_max_ models were used to determine dose-response.

**Results:**

38 PSAC, 133 SAC, and 196 adults were enrolled. In adults, predicted CRs increased with ascending doses of albendazole, with a CR of 74.9% (95% confidence interval [CI], 55.6%–87.7%) in the 800-mg arm. Observed CRs increased with ascending doses of albendazole reaching a maximum of 94.1% (95% CI, 80.3%–99.3%). In SAC, the predicted dose-response curve increased marginally, with CRs ranging from 64.0% in the 200-mg arm to 76.0% in the 800-mg arm. Sample size in PSAC was considered too small to derive meaningful conclusions. 10.7% and 5.1% of participants reported any adverse event at 3 hours and 24 hours post-treatment, respectively.

**Conclusions:**

A single 800-mg albendazole dose provides higher efficacy against hookworm and is well tolerated in adults and should be considered for community-based strategies targeting adults. For PSAC and SAC, current recommendations suffice.

**Clinical Trials Registration:**

NCT03527745.

## Teaser

A higher dose of albendazole in adults for the treatment of hookworm infection provides increased cure rates with high tolerability, whereas an increase in dose does not provide additional benefit in children.

*Necator americanus* or *Ancylostoma duodenale*, two types of hookworm, affect approximately 500 million individuals worldwide [1, 2]. As a soil-transmitted helminth (STH), hookworm is most common in tropical and subtropical settings where larvae are able to survive in moist, warm soil.

Hookworm accounted for approximately 845 000 disability-adjusted life-years lost in 2017, with an estimated economic loss of more than $100 billion [3, 4]. The hookworm can cause skin irritation when it enters through the skin, acute lung inflammation and difficulty breathing when larvae migrate through the pulmonary system, and gastrointestinal issues such as blood loss, diarrhea, and abdominal pain [5, 6]. Morbidity is highly correlated with infection intensity, and more serious infections can cause anemia from blood loss [3, 7].

Albendazole is one of two benzimidazoles currently on the World Health Organization (WHO) Model List of Essential Medicines as a anthelmintic against intestinal STHs [8]. Developed in 1975 and licensed less than a decade later for human use, albendazole is part of current global control strategies [9, 10]. The mainstay of control is based on targeted preventative chemotherapy (PC) in preschool-aged children (PSAC), school-aged children (SAC), and women of reproductive age (WRA) given annually/biannually using either 400 mg of albendazole or 500 mg of mebendazole [10]. In 2012, the WHO published targets for 75% of coverage with PC in PSAC and SAC in all endemic countries and regular treatment of about 600 million PSAC and SAC who are in need of treatment by 2020 [11]. In 2018, additional targets were developed with the aim of STH control by 2030, including achieving and maintaining elimination in PSAC and SAC and expanding control programs to WRA [12].

Though PC with benzimidazoles has been successfully used as a cornerstone of control programs, efficacy against hookworm remains moderate (cure rates [CRs] of 79.5% for 400 mg albendazole and 32.5% for 500 mg of mebendazole) [13]. Several studies have shown that multiple doses of albendazole over an increased number of days have higher efficacy; however, this strategy could prove difficult during mass drug administration (MDA) campaigns [14–18]. A single dose that is greater than 400 mg may be more efficacious and effective at curing hookworm infection, though an optimal dose has not been identified, and limited evidence on the efficacy and safety for PSAC and adults is available. The novel aim of this trial is to characterize the dose-response relationship for the efficacy and safety of albendazole against hookworm in PSAC, SAC, and adults.

## METHODS

### Study Design, Area, and Population

A phase 2, parallel, randomized, controlled trial was conducted in 21 villages surrounding the town of Rubino in southeastern Côte d’Ivoire from 5 January 2019 to 17 April 2019. PSAC aged 2–5 years, SAC aged 6–12 years, and adults aged ≥21 years were invited to participate in the trial.

### Ethical Considerations

Ethical permission from the Comité National d’Éthique des Sciences de la Vie et de la Santé in Côte d’Ivoire was issued on 3 July 2018, while the Northwestern and Central Switzerland Ethics Committee provided ethical clearance on 20 July 2018. Written informed consent was obtained from adult participants and from the parents/guardians of participating children after they had attended a community-wide information meeting. Additionally, SAC provided written consent. 

### Eligibility Criteria

Participants were eligible for the trial if they were willing to provide informed consent and two stool samples. Only participants with hookworm infection intensity of ≥24 eggs per gram (EPG) of stool were included. Those with acute or uncontrolled systemic illness (eg, severe anemia defined as hemoglobin <8.0 g/dL, infection, clinical malaria with fever) as assessed by a medical doctor, those who had received anthelmintics within the previous four weeks, those with allergies to benzimidazoles, and women who were pregnant or breastfeeding were excluded.

### Randomization and Treatment

A computer-generated, stratified block randomization code was generated by the trial statistician who was not involved in participation recruitment, treatment administration, or outcome assessment. The randomization list with varying blocks of three and six for PSAC or five and ten for SAC and adults was stratified by baseline hookworm infection intensity (either light or moderate/heavy) per WHO guidelines [19]. After a lengthy period of recruitment, it was decided to eliminate the placebo group for the PSAC cohort. PSAC were randomized 1:1:1 to albendazole at 200 mg, 400 mg, or 600 mg. SAC and adults were randomized 1:1:1:1:1 to albendazole at 200 mg, 400 mg, 600 mg, or 800 mg or placebo. Outcome assessors were blinded to treatment allocation. Participants were potentially aware of the treatment arm that they were assigned due to the different number of tablets provided by unblinded study investigators.

### Field and Laboratory Procedures

After a census in each community was conducted, children and adults in the appropriate age groups were asked to provide two stool samples. From each sample, duplicate Kato-Katz thick smears (each 41.7 mg) were prepared and examined for hookworm, *Trichuris trichiura*, and *Ascaris lumbricoides* eggs by two independent, trained technicians using light microscopes [20]. A third technician conducted an independent quality control for 10% of the slides. Results were considered correct if no difference in presence/absence of each helminth or egg counts were ±10 eggs for counts ≤100 eggs or ±20% for counts >100 eggs (for each species) [21]. Slides with incorrect results were re-read until a consensus was met.

Participants were invited for a clinical examination where individuals were physically examined, questioned for clinical symptoms, and underwent a series of rapid tests for malaria and pregnancy. Additionally, individuals were asked to provide a venous blood sample to assess complete blood count and hepatic/renal function.

Active adverse event (AE) reporting was conducted 3 hours, 24 hours, and 14–21 days post-treatment. At 14–21 days post-treatment, enrolled participants were asked to provide two stool samples for quadruplicate Kato-Katz smears, which were collected and analyzed as described above. At the end of the trial, all individuals who provided any stool sample and remained positive for any STH infection were treated with 400 mg of albendazole per WHO recommendations [10].

### Outcome Measures

CR against hookworm infection at 14–21 days post-treatment was the primary outcome. Secondary outcomes were egg reduction rates (ERRs) against hookworm, CRs and ERRs against other STHs, and drug safety.

### Sample Size

Multiple simulations were conducted to justify the required sample size. Assumed CRs against hookworm for placebo and albendazole at 200 mg, 400 mg, 600 mg, and 800 mg were 2.5%, 30%, 50%, 70%, and 80%, respectively, while loss to follow-up was estimated at 10%. Simulations indicated that 40 participants per arm would be sufficient to predict the dose-response curve with a median precision, defined as one-half the length of the corresponding confidence band of approximately 10 percentage points [22].

### Statistical Analyses

Data were collected on paper forms by trained personnel and entered twice into a database (Microsoft Access 2010, Wallisellen, Switzerland). Data were cross-checked using Beyond Compare 4 (Scooter Software, Madison, WI), and discrepancies were corrected using the original data. Analysis was performed using Stata, version 15 (StataCorp, College Station, TX) and R software, version 3.5.1 (www.r-project.org).

An analysis set of all randomized participants who provided any follow-up data was used to perform an available-case analysis. CRs were calculated as the percentage of egg-positive participants at baseline who become egg-negative post-treatment. EPG was calculated using the mean egg counts from the quadruplicate Kato-Katz thick smears and multiplying by a factor of 24. ERRs were calculated using geometric mean egg counts with the following formula:


$$\begin{array}{l} ERR=1-\frac{\frac{1}{n}\;e^{\sum \log\left(EPG_{follow-up}+1\right)}-1}{\frac{1}{n}\;e^{\sum \log\left(EPG_{baseline}+1\right)}-1} \end{array}$$

The 95% confidence intervals (CIs) for ERR point estimates were calculated using a bootstrap resampling method with 5000 replicates. ERRs were not calculated for infections of *T. trichiura* and *A. lumbricoides* because too few infections were observed. To predict dose-response curves in terms of CRs and ERRs, hyperbolic *E*_max_ models were fitted using the R software DoseFinding package.

The number and proportion of AEs and participants reporting AEs were descriptively summarized before and after treatment.

## RESULTS

### Baseline Characteristics

**Figure 1.**
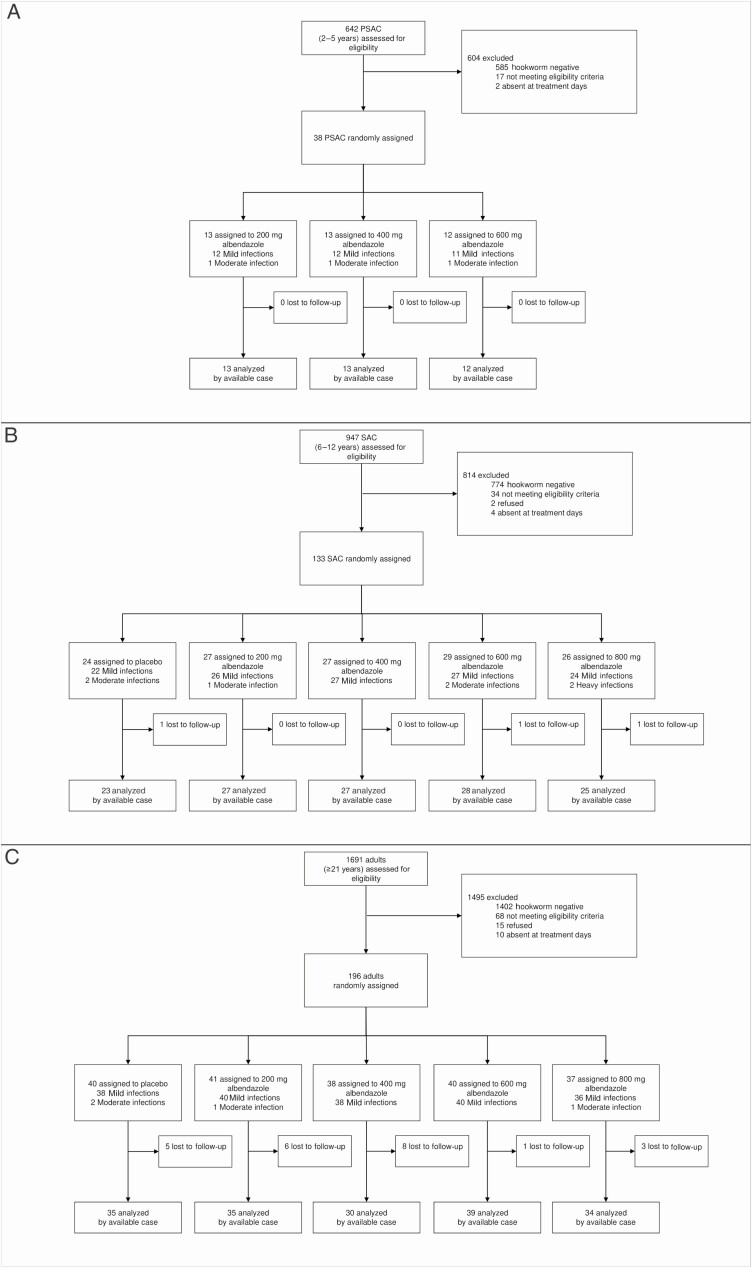
Trial flow charts. *A*, PSAC participant flow chart. *B*, SAC participant flow chart. *C*, Adults participant flow chart. Abbreviations: PSAC, preschool-aged children; SAC, preschool-aged children.

Figure 1 shows participant flow charts for each age cohort. A total of 642 PSAC, 947 SAC, and 1691 adults were screened for eligibility. Of those screened, 57 PSAC, 173 SAC, and 289 adults were positive for hookworm infection. Hence, despite exhaustive screening efforts, target sample sizes for PSAC (120) and SAC (200) were not reached. Based on eligibility criteria of ≥24 EPG, 50 PSAC, 164 SAC, and 256 adults were invited to participate in clinical examination. There were 12 PSAC, 31 SAC, and 60 adults who refused to participate, were excluded based on eligibility criteria on the day of treatment, or were absent.

On treatment day, 38 PSAC, 133 SAC, and 196 adults were randomized to either three or five treatment arms (Figure 1). At 14–21 days post-treatment, 3 SAC (2.3%) and 23 adults (11.7%) did not provide any stool samples or were absent; all PSAC provided stool samples at follow-up.

Baseline demographic and parasitological characteristics of PSAC, SAC, and adults included are presented in Table 1. The proportions of females were lower in the 200-mg group in PSAC and higher in the 200-mg arm in SAC and adults. Other baseline characteristics were balanced. The majority of infections were of mild intensity (ranging from 92% to 100% across arms) in all three age cohorts; only 3 PSAC, 5 SAC, and 4 adults had moderate-intensity infections, and there were no high-intensity infections. Only 1 PSAC, 6 SAC, and 2 adults were coinfected with *T. trichiura*, and 2 PSAC, 3 SAC, and 1 adult were coinfected with *A. lumbricoides*.

**Table 1.** Baseline Characteristics of Participants

	Preschool-aged Children			School-aged Children					Adults				
Characteristic	Albendazole, 200 mg (n = 13)	Albendazole, 400 mg (n = 13)	Albendazole, 600 mg (n = 12)	Placebo (n = 24)	Albendazole, 200 mg (n = 27)	Albendazole, 400 mg (n = 27)	Albendazole, 600 mg (n = 29)	Albendazole, 800 mg (n = 26)	Placebo (n = 40)	Albendazole, 200 mg (n = 41)	Albendazole, 400 mg (n = 38)	Albendazole, 600 mg (n = 40)	Albendazole, 800 mg (n = 37)
Age, mean (SD), y	3.8 (1.2)	3.8 (1.3)	3.8 (1.1)	9.1 (2.2)	9.1 (1.9)	9.8 (1.9)	9.2 (2.2)	9.4 (2.3)	35.4 (10.3)	39.8 (12.2)	36.3 (12.2)	38.2 (11.5)	35.7 (12.0)
Females	5 (38%)	9 (69%)	8 (67%)	8 (33%)	13 (48%)	8 (30%)	5 (17%)	7 (27%)	7 (18%)	16 (39%)^a^	7 (18%)	7 (18%)	9 (24%)
Weight, mean (SD), kg	15.5 (3.6)	13.8 (3.8)	14.4 (2.9)	26.0 (5.4)	25.7 (5.5)	27.7 (6.8)	24.8 (7.0)	27.9 (10.3)	59.7 (9.9)	61.3 (9.5)	61.6 (9.3)	62.7 (8.0)^b^	59.7 (8.8)
Height, mean (SD), cm	98.8 (12.3)	98.1 (9.5)	96.1 (9.5)	132.6 (13.2)	127.2 (12.7)	129.8 (13.1)	123.9 (18.2)	137.5 (39.7)	165.8 (10.7)^a^	166.6 (10.7)	168.1 (7.5)	168.3 (10.0)^a^	165.2 (9.3)^a^
Hookworm													
Median EPG (interquartile range)	216 (150–618)	126 (60–540)	252 (111–423)	183 (78–450)	126 (72–336)	138 (78–246)	156 (84–528)	153 (66–366)	57 (96–318)	126 (54–444)	96 (54–264)	189 (63–570)	114 (84–216)
EPG geometric mean	251.1	178.4	238.8	209.1	166.7	162.1	219.5	226.4	136.5	147.9	116.3	198.4	151.8
Infection intensity													
Mild (1–1999 EPG)	12 (92%)	12 (92%)	22 (92%)	22 (92%)	26 (96%)	27 (100%)	27 (93%)	24 (92%)	38 (95%)	40 (98%)	38 (100%)	40 (100%)	36 (97%)
Moderate (2000–3999 EPG)	1 (8%)	1 (8%)	2 (8%)	2 (8%)	1 (4%)	0 (0%)	2 (7%)	0 (0%)	2 (5%)	1 (2%)	0 (0%)	0 (0%)	1 (3%)
Heavy (≥4000 EPG)	0 (0%)	0 (0%)	0 (0%)	0 (0%)	0 (0%)	0 (0%)	0 (0%)	2 (8%)	0 (0%)	0 (0%)	0 (0%)	0 (0%)	0 (0%)
*Trichuris trichiura*	0	0	1	2	2	0	0	2	1	0	0	0	1
*Ascaris lumbricoides*	1	0	1	1	1	0	1	0	0	0	0	1	0

Abbreviations: EPG, eggs per gram; SD, standard deviation. ^a^Missing data from 1 participant. ^b^Missing data from 2 participants.

### Efficacy

**Figure 2.**
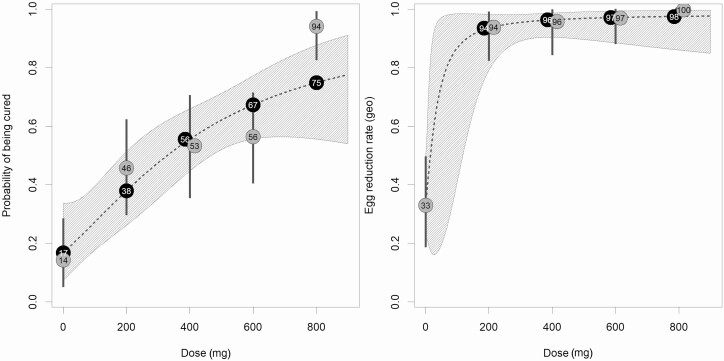
Cure rates (CRs) and egg reduction rates (ERRs) of albendazole in adults predicted by the hyperbolic *E*_max_ model. Dotted lines represent the dose-response curve, and the hatched area corresponds to the 95% confidence band. White numbers present the predicted CRs and ERRs for the investigated doses. Gray circles with black numbers represent the observed dose group CRs, and the gray vertical lines correspond to the 95% confidence intervals. Abbreviation: geo, geometric.

For adults, CRs and ERRs of each arm against hookworm infection are presented in Table 2; results for PSAC and SAC can be found in Supplementary Table 1. The *E*_max_ model predicted a maximum CR of 97.2% (placebo adjusted, 80.4%) and a half maximal-effect dose (ED_50_) on logit scale at 724 mg in the adult cohort (314 mg on probability scale). The dose-response curve showed no plateau within the observed dose range. The predicted CRs were 16.8% (95% CI, 7.4%–33.8%), 37.9% (95% CI, 26.2%–51.3%), 55.6% (95% CI, 44.7%–66.0%), 67.3% (95% CI, 55.6%–77.2%), and 74.9% (95% CI, 55.6%–87.7%) for placebo, 200 mg, 400 mg, 600 mg, and 800 mg, respectively (Figure 2). Likewise, observed CRs increased with ascending doses of albendazole and reached a maximum of 94.1% (95% CI, 80.3%–99.3%). In terms of ERRs, the *E*_max_ predicted ERR at the first investigated dose of 200 mg was 93.5% (95% CI, 79.1%–98.2%) and increased to 97.5% (95% CI, 86.0%–99.6%) in the 800-mg albendazole arm (Figure 2). Observed and predicted ERRs differed only slightly across all treatment arms.

**Table 2.** Observed and Predicted Cure Rates and Egg Reduction Rates Against Hookworm of Adults at 3 Weeks Follow-up

Result	Placebo	Albendazole, 200 mg	Albendazole, 400 mg	Albendazole, 600 mg	Albendazole, 800 mg
Positive before treatment	35	35	30	39	34
Cured after treatment	5	16	16	22	32
Observed CR (95% CI)	14.3 (4.8 to 30.3)	45.7 (28.8 to 63.4)	53.3 (34.3 to 71.7)	56.4 (39.6 to 72.2)	94.1 (80.3 to 99.3)
Predicted CR (95% CI)	16.8 (7.4 to 33.8)	37.9 (26.2 to 51.3)	55.6 (44.7 to 66.0)	67.3 (55.6 to 77.2)	74.9 (55.6 to 87.7)
EPG geometric mean					
Baseline	135.9	132.3	118.6	204.1	139.6
3 weeks follow-up	91.1	8.2	5.0	6.2	0.2
Observed ERR (95% CI)	33.0 (−27.1 to 67.3)	93.8 (87.0 to 97.3)	95.8 (90.2 to 98.3)	97.0 (92.5 to 98.9)	99.8 (99.5 to 100.0)
Predicted ERR (95% CI)	33.0 (19.6 to 49.9)	93.5 (79.1 to 98.2)	96.4 (90.4 to 98.7)	97.1 (88.4 to 99.3)	97.5 (86.0 to 99.6)
EPG arithmetic mean					
Baseline	348.3	325.0	242.0	414.9	275.6
3 weeks follow-up	478.6	63.9	32.2	69.5	2.1
Observed ERR (95% CI)	−37.4 (−137.1 to 44.6)	80.3 (58.1 to 91.8)	86.7 (75.3 to 94.0)	83.3 (68.0 to 92.7)	99.2 (97.6 to 100.0)

Abbreviations: CI, confidence interval; CR, cure rate; EPG, eggs per gram; ERR, egg reduction rate.

In SAC, observed CRs and ERRs against hookworm showed a less pronounced dose-response effect compared with adults. The increase in terms of CR after 400 mg was small. Observed CRs ranged from 63.0% (95% CI, 42.4%–80.6%) in the 200-mg arm to 76.0% (95% CI, 54.9%–90.6%) in the 800-mg arm. The observed CR in the placebo group was 43.5% (95% CI, 23.2%–65.5%). Geometric ERRs were high in all arms. The number of eligible PSAC was far below the anticipated sample size, and results are only descriptively analyzed. Observed CRs were 69.2%, 61.5%, and 83.3% and geometric mean–based ERRs were 99.0%, 96.2%, and 99.6% for 200 mg, 400 mg, and 600 mg of albendazole, respectively.

Supplementary Table 2 presents the proportions of participants cured of hookworm infection within each treatment arm by sex.

There were very few coinfections with *T. trichiura* and/or *A. lumbricoides* (9 and 5 participants, respectively). Only two participants remained coinfected with mild infection at three weeks post-treatment, and one participant with trichuriasis was lost to follow-up.

### Safety

At baseline, 38 PSAC, 130 SAC, and 173 adults were questioned for symptoms. Among these, 34 participants (10.0%) reported a total of 43 mild symptoms at baseline, the majority of which were reported by adults. The most common reported symptoms at baseline were abdominal pain (9.2%), headache (6.4%), and itching (4.7%) in adults.

At three hours post-treatment, 34 PSAC, 118 SAC, and 137 adults were interviewed. From these, 2 PSAC, 11 SAC, and 18 adults reported at least one AE (10.7%). The most common AEs reported at 3 hours follow-up were abdominal pain (10.1% of SAC and 5.8% of adults) and headache (10.0% in adults, 5.9% in PSAC, and 2.2% in SAC).

A total of 28 PSAC, 89 SAC, and 120 adults were interviewed at 24 hours follow-up; 12 reported experiencing any AE (5.1%). Abdominal pain was the most common reported AE at the 24-hour examination, reported by 2.2% of SAC and 5.0% of adults.

The number of AEs reported and number of participants reporting any AE by each treatment arm can be found in Table 3. A more detailed report of symptom-specific AEs can be found in Supplementary Table 3.

**Table 3.** Number of Adverse Events and Number of Participants Reporting Adverse Events

		Preschool-aged Children				School-aged Children						Adults					
Time Point	Number of	Albendazole, 200 mg	Albendazole, 400 mg	Albendazole, 600 mg	Total	Placebo	Albendazole, 200 mg	Albendazole, 400 mg	Albendazole, 600 mg	Albendazole, 800 mg	Total	Placebo	Albendazole, 200 mg	Albendazole, 400 mg	Albendazole, 600 mg	Albendazole, 800 mg	Total
Before treatment	Symptoms	0	0	1	1	1	0	0	1	1	3	6	10	11	5	7	39
	Participants	0	0	1	1	1	0	0	1	1	3	5	8	8	4	5	30
3 hours after treatment	Adverse events	1	0	1	2	2	3	2	1	3	11	2	4	5	4	5	20
	Participants	1	0	1	2	2	3	2	1	3	11	2	4	5	3	4	18
24 hours after treatment	Adverse events	0	0	0	0	0	0	2	1	0	3	1	2	2	5	1	11
	Participants	0	0	0	0	0	0	2	1	0	3	1	2	2	3	1	9

## DISCUSSION

Currently, there are several drugs approved by the WHO for the treatment/control of STH infections, though albendazole and mebendazole are the most common due to their ease of administration (single dose regardless of weight) and low cost [8, 23]. Albendazole, being more efficacious than mebendazole against hookworm, is a cornerstone of PC programs in both moderate/high and low STH transmission settings; however, the standard dose of 400 mg varies in efficacy [13]. Factors such as infection intensity, coinfection with other STHs, and species type have all been proposed as determinants of the variability of albendazole efficacy [13, 15, 23–25].

The results of this trial confirm that at a recommended 400-mg dose, albendazole is moderately efficacious against hookworm infection in PSAC, SAC, and adults (CRs of 61.5%, 74.1%, and 53.3%, respectively). In a recent systematic review, a comparable CR of 400 mg of albendazole against hookworm was found to be 70.5% (95% CI, 54.5%–82.6%) in trials conducted between 2000 and 2016 [13]. A published review of the literature until 1999 showed that efficacy of a single dose of 400 mg of albendazole was 77.7%, specifically 91.8% against *A. duodenale* and 75.0% against *N. americanus* [15]. In the same review, the efficacy of 200 mg of albendazole was found to be significantly lower (70.0%) in older children and adults, which is clearly confirmed in this trial for adults [15].

Remarkably, a higher dose of albendazole has increased efficacy, with a single dose of 800 mg of albendazole having a CR of 94.1% in adults. In PSAC and SAC, efficacy of albendazole remained moderate in all intervention arms; moreover, a slight increase in efficacy was seen in PSAC with ascending doses, though results should be interpreted carefully due to the small number of PSAC in each arm. No well-controlled trials were found specifically for PSAC or SAC; however, CRs for 200 mg, 600 mg, and 800 mg that were reported in a systematic review were within the ranges reported in this trial for the respective doses [15].

The greatest limitation of this trial was the poor recruitment of children. Only 24% and 67% of needed PSAC and SAC, respectively, were recruited into the trial. Hence, as mentioned before, it was difficult to identify the optimal dose of albendazole in these cohorts. The generalizability of the results are also limited to settings where infection intensity and coinfection with other STHs are low; evidence suggests that albendazole’s efficacy is affected by these factors [13, 23]. Since the diagnostic method used did not differentiate between the two common hookworm species, efficacy results may not be applicable to settings where *A. duodenale* is the predominant species, as *N. americanus* is most prevalent in sub-Saharan Africa [6, 16, 26, 27].

Current recommendations of a single dose of a benzimidazole administered annually or biannually to SAC may not be enough to eliminate helminthiasis. Recent models have shown that PC that targets only SAC will not eliminate hookworm infections and that to interrupt hookworm transmission, community-based PC that also targets adults who are the main reservoir of hookworm transmission, which was confirmed in this trial, is needed [25, 28, 29]. Community-based MDA was shown to reduce the infection rate of hookworm by 91% in a study carried out from 2012 to 2013 in the Republic of Congo [30]. A recent clinical trial conducted in Kenya showed community-based treatment strategies to be more effective than school-based treatment in reducing hookworm prevalence; moreover, the costs were equitable [31]. Currently, a series of trials are being conducted in one Asian and two African settings to evaluate the feasibility of hookworm and other STH transmission interruption with community-based MDA [32].

Community-based MDA aligns with the 2030 targets of the NTD Roadmap, which plans to expand control programs to WRA, who are at risk of iron-deficiency anemia [12, 23]. Evidence from this trial can have a direct impact on the program strategies for the control and elimination of hookworm infection. Given the remarkable higher efficacy observed, increasing the dose of albendazole for adults targeted by community-based MDA may aid in the interruption of transmission.

The estimated cost of one albendazole tablet is $0.018 with an additional 10% for overhead costs [33]. Recent estimates have shown that the average per-person cost of annual treatment at the community level is $0.68, which could be reduced during scale-up to $0.33 if coverage remains high [31]. These estimates are within the reported range of costs ($0.33–$0.70) for treating an infected individual reported by the WHO in 2017 [10]. Integrating an additional albendazole tablet into program strategies for treatment of adults would not drive costs outside of current ranges, though more evidence on the cost-effectiveness of two tablets of 400 mg albendazole for adults is needed.

In conclusion, this trial provides direct evidence on the optimal dose of albendazole for treatment of hookworm infection in low-transmission settings. Though sample sizes in SAC and PSAC were lower than anticipated, our findings support use of the currently recommended albendazole doses since increasing doses revealed no benefit in these age groups. However, we observed a considerably higher efficacy with administration of 800 mg of albendazole in the adult cohort. Strategies to integrate an 800-mg dose of albendazole to adults during community-based MDA should therefore be explored.

## Supplementary Data

Supplementary materials are available at *Clinical Infectious Diseases* online. Consisting of data provided by the authors to benefit the reader, the posted materials are not copyedited and are the sole responsibility of the authors, so questions or comments should be addressed to the corresponding author.

ciaa989_suppl_Supplementary_Material

## Notes

***Author contributions.*** C. P., J. T. C., J. H., and J. K. designed the study. C. P., J. T. C., D. H., and J. K. performed the study. C. P., J. H., and J. K. analyzed and interpreted the data. C. P. and J. K. wrote the initial draft of the manuscript. J. T. C., D. H., and J. H. revised, read, and approved the final version of the manuscript.

***Acknowledgments.*** The authors thank all participating children and adults from the 21 villages. Additional thanks to the field workers and the field/laboratory teams from the Centre Suisse de Recherches Scientifiques in Abidjan, Côte d’Ivoire, for their exemplary conduct in this trial.

***Financial support.*** This work was supported by the Bill and Melinda Gates Foundation (grant OPP1153928).

***Potential conflicts of interest.*** The authors: No reported conflicts of interest. All authors have submitted the ICMJE Form for Disclosure of Potential Conflicts of Interest.
