# Supplementary material for: Efficacy and Safety of Albendazole in Hookworm-infected Preschool-aged Children, School-aged Children, and Adults in Côte d’Ivoire: A Phase 2 Randomized, Controlled Dose-finding Trial
Source: Clin Infect Dis. 2020 Jul 15;73(2):e494–502. doi: 10.1093/cid/ciaa989 (PMC8282316; doi:10.1093/cid/ciaa989)
Supplement: ciaa989_suppl_Supplementary_Material [file ciaa989_suppl_supplementary_material.docx]

**Supplementary Table 1:** Cure rates and egg reduction rates against hookworm of PSAC and SAC at 3 weeks follow-up. Abbreviations: ALB, albendazole; CI, confidence interval; CR, cure rate; EPG, eggs per gram; ERR, egg reduction rate; PLAC, placebo; PSAC, preschool-aged children; SAC, school-aged children.

|  | | **PSAC** | | | **SAC** | | | | |
| --- | --- | --- | --- | --- | --- | --- | --- | --- | --- |
|  | | **ALB 200 mg** | **ALB 400 mg** | **ALB 600 mg** | **PLAC** | **ALB 200 mg** | **ALB 400 mg** | **ALB 600 mg** | **ALB 800 mg** |
| Positive before treatment | | 13 | 13 | 12 | 23 | 27 | 27 | 28 | 25 |
| Cured after treatment | | 9 | 8 | 10 | 10 | 17 | 20 | 18 | 19 |
| Observed CR  [95% CI] | | 69.2  [38.6, 90.9] | 61.5  [31.6, 86.1] | 83.3  [51.6, 97.9] | 43.5  [23.2, 65.5] | 63.0  [42.4, 80.6] | 74.1  [53.7, 88.9] | 64.3  [44.1, 81.4] | 76.0  [54.9, 90.6] |
| Predicted CR  [95% CI] | |  |  |  | 43.4  [25.2, 63.6] | 64.0  [46.4, 78.5] | 68.9  [59.1, 77.2] | 71.0  [59.8, 80.1] | 72.1  [57.7, 83.1] |
| EPG geometric mean | |  |  |  |  |  |  |  |  |
|  | Baseline | 251.1 | 178.4 | 238.8 | 229.5 | 166.7 | 162.1 | 200.1 | 234.9 |
|  | 3 weeks follow-up | 2.6 | 6.8 | 0.9 | 21.5 | 3.9 | 1.6 | 3.4 | 3.8 |
|  | Observed ERR [95% CI] | 99.0  [94.7, 99.9] | 96.2  [83.5, 99.4] | 99.6  [98.5, 100] | 90.6  [72.3, 97.3] | 97.7  [93.5, 99.3] | 99.0  [97.4, 99.7] | 98.3  [95.4, 99.5] | 98.4  [95.9, 99.6] |
|  | Predicted ERR [95% CI] |  |  |  | 90.6  [70.4, 97.5] | 97.9  [80.3, 99.8] | 98.3  [92.9, 99.6] | 98.5  [91.1, 99.7] | 98.5  [88.2, 99.8] |
| EPG arithmetic mean | |  |  |  |  |  |  |  |  |
|  | Baseline | 528.5 | 422.8 | 398.0 | 500.1 | 343.3 | 262.7 | 398.8 | 1102.6 |
|  | 3 weeks follow-up | 42.8 | 275.5 | 7.2 | 347.8 | 150.7 | 13.6 | 30.2 | 524.9 |
|  | Observed ERR [95% CI] | 91.9  [72.2, 99.8] | 34.8  [-1.5, 94.1] | 98.2  [93.6, 100] | 30.4  [-6.4, 68.0] | 56.1  [19.0, 96.3] | 94.8  [89.5, 98.6] | 92.4  [83.2, 97.0] | 52.4  [32.9, 91.6] |

**Supplementary Table S2:** Proportion of participants cured by treatment arm and sex in PSAC, SAC and adults. Abbreviations: ALB, albendazole; F, female; M, male; na, not applicable; PLAC, placebo; PSAC, preschool-aged children; SAC, school-aged children.

|  |  | **PSAC** | | **SAC** | | **Adults** | |
| --- | --- | --- | --- | --- | --- | --- | --- |
| **PLAC** | **F** | na |  | 3/8 | (37.5%) | 1/7 | (14.3%) |
|  | **M** | na |  | 7/15 | (46.7%) | 5/35 | (14.3%) |
| **ALB**  **200 mg** | **F** | 3/5 | (60.0%) | 8/13 | (61.5%) | 9/14 | (64.3%) |
|  | **M** | 6/8 | (75.0%) | 9/14 | (64.3%) | 14/21 | (33.3%) |
| **ALB**  **400 mg** | **F** | 5/9 | (55.6%) | 5/8 | (62.5%) | 2/4 | (50.0%) |
|  | **M** | 3/4 | (75.0%) | 15/19 | (79.0%) | 14/26 | (53.9%) |
| **ALB**  **600 mg** | **F** | 7/8 | (87.5%) | 2/5 | (40.0%) | 4/7 | (57.1%) |
|  | **M** | 3/4 | (75.0%) | 16/23 | (69.6%) | 18/32 | (56.3%) |
| **ALB**  **800 mg** | **F** | na |  | 6/7 | (85.7%) | 9/9 | (100.0%) |
|  | **M** | na |  | 13/18 | (72.2%) | 23/25 | (92.0%) |

**Supplementary Table S3:** Reported number of participants experiencing adverse events by treatment arm among PSAC, SAC, and adults questioned. Abbreviations: ALB, albendazole; PLAC, placebo; PSAC, preschool-aged children; SAC, school-aged children.

|  | | **PSAC** | | | **SAC** | | | | | **Adults** | | | | |  |
| --- | --- | --- | --- | --- | --- | --- | --- | --- | --- | --- | --- | --- | --- | --- | --- |
| **Adverse event** | | **ALB 200 mg** | **ALB 400 mg** | **ALB 600 mg** | **PLAC** | **ALB**  **200 mg** | **ALB 400 mg** | **ALB 600 mg** | **ALB 800 mg** | **PLAC** | **ALB**  **200 mg** | **ALB 400 mg** | **ALB 600 mg** | **ALB 800 mg** | |
| **Before treatment** | |  |  |  |  |  |  |  |  |  |  |  |  |  | |
|  | Headache | 0/13 (0.0) | 0/13 (0.0) | 0/12  (0.0) | 0/23  (0.0) | 0/27  (0.0) | 0/27 (0.0) | 0/28  (0.0) | 0/25  (0.0) | 2/35  (5.7) | 2/35  (5.7) | 2/30  (6.7) | 3/39  (7.7) | 2/34  (5.9) | |
|  | Abdominal pain | 0/13 (0.0) | 0/13 (0.0) | 0/12  (0.0) | 0/23  (0.0) | 0/27  (0.0) | 0/27 (0.0) | 1/28  (3.6) | 0/25  (0.0) | 3/35  (8.6) | 6/35  (17.1) | 4/30  (13.3) | 1/39  (2.6) | 2/34  (5.9) | |
|  | Nausea | 0/13 (0.0) | 0/13 (0.0) | 0/12  (0.0) | 0/23  (0.0) | 0/27  (0.0) | 0/27 (0.0) | 0/28  (0.0) | 0/25  (0.0) | 0/35  (0.0) | 0/35  (0.0) | 0/30  (0.0) | 0/39  (0.0) | 0/34  (0.0) | |
|  | Vomiting | 0/13 (0.0) | 0/13 (0.0) | 0/12  (0.0) | 0/23  (0.0) | 0/27  (0.0) | 0/27 (0.0) | 0/28  (0.0) | 0/25  (0.0) | 0/35  (0.0) | 0/35  (0.0) | 0/30  (0.0) | 0/39  (0.0) | 0/34  (0.0) | |
|  | Diarrhea | 0/13 (0.0) | 0/13 (0.0) | 1/12  (8.3) | 1/23  (4.4) | 0/27  (0.0) | 0/27 (0.0) | 0/28  (0.0) | 0/25  (0.0) | 0/35  (0.0) | 1/35  (2.86) | 2/30  (6.7) | 0/39  (0.0) | 1/34 (2.9) | |
|  | Itching | 0/13 (0.0) | 0/13 (0.0) | 0/12  (0.0) | 0/23  (0.0) | 0/26  (0.0) | 0/27 (0.0) | 0/27  (0.0) | 1/24  (4.2) | 1/34 (3.0) | 1/35  (2.86) | 3/30  (10.0) | 1/39  (2.6) | 2/33 (6.1) | |
| **3 hr after treatment** | | |  |  |  |  |  |  |  |  |  |  |  |  | |
|  | Headache | 1/12  (8.3) | 0/10  (0.0) | 1/12  (8.3) | 1/21  (4.8) | 1/24  (4.2) | 0/25  (0.0) | 0/26  (0.0) | 0/22  (0.0) | 2/31  (6.5) | 3/28  (10.7) | 4/21 (19.1) | 2/31  (6.5) | 1/26  (3.9) | |
|  | Abdominal pain | 0/12  (0.0) | 0/10  (0.0) | 0/12  (0.0) | 1/21  (4.8) | 2/24  (8.3) | 2/25  (8.0) | 1/26  (3.9) | 3/22  (13.6) | 0/31  (0.0) | 1/28  (3.6) | 1/21 (4.7) | 2/31  (6.5) | 3/26  (11.5) | |
|  | Nausea | 0/12  (0.0) | 0/10  (0.0) | 0/12  (0.0) | 0/21  (0.0) | 0/24  (0.0) | 0/25  (0.0) | 0/26  (0.0) | 0/22  (0.0) | 0/31 (0.0) | 0/28  (0.0) | 0/21  (0.0) | 0/31  (0.0) | 1/26  (3.9) | |
|  | Vomiting | 0/12  (0.0) | 0/10  (0.0) | 0/12  (0.0) | 0/21  (0.0) | 0/24  (0.0) | 0/25  (0.0) | 0/26  (0.0) | 0/22  (0.0) | 0/31 (0.0) | 0/28  (0.0) | 0/21  (0.0) | 0/31  (0.0) | 0/26  (0.0) | |
|  | Diarrhea | 0/12  (0.0) | 0/10  (0.0) | 0/12  (0.0) | 0/21  (0.0) | 0/24  (0.0) | 0/25  (0.0) | 0/26  (0.0) | 0/22  (0.0) | 0/31 (0.0) | 0/28  (0.0) | 0/21  (0.0) | 0/31  (0.0) | 0/26  (0.0) | |
|  | Itching | 0/12  (0.0) | 0/10  (0.0) | 0/12  (0.0) | 0/21  (0.0) | 0/24  (0.0) | 0/25  (0.0) | 0/26  (0.0) | 0/22  (0.0) | 0/31 (0.0) | 0/28  (0.0) | 0/21  (0.0) | 0/31  (0.0) | 0/26  (0.0) | |
|  | Serious adverse event | 0/12  (0.0) | 0/10  (0.0) | 0/12  (0.0) | 0/21  (0.0) | 0/24  (0.0) | 0/25  (0.0) | 0/26  (0.0) | 0/22  (0.0) | 0/31 (0.0) | 0/28  (0.0) | 0/21  (0.0) | 0/31  (0.0) | 0/26  (0.0) | |
| **24 hr after treatment** | | |  |  |  |  |  |  |  |  |  |  |  |  | |
|  | Headache | 0/8 (0.0) | 0/9 (0.0) | 0/11 (0.0) | 0/15  (0.0) | 0/18  (0.0) | 0/23  (0.0) | 0/17  (0.0) | 0/16  (0.0) | 0/24  (0.0) | 0/24  (0.0) | 0/21  (0.0) | 1/27  (3.7) | 0/24  (0.0) | |
|  | Abdominal pain | 0/8 (0.0) | 0/9 (0.0) | 0/11 (0.0) | 0/15  (0.0) | 0/18  (0.0) | 2/23  (8.7) | 0/17  (0.0) | 0/16  (0.0) | 0/24  (0.0) | 1/24  (4.2) | 2/21  (9.5) | 3/27  (11.1) | 0/24  (0.0) | |
|  | Nausea | 0/7 (0.0) | 0/6 (0.0) | 0/8 (0.0) | 0/11 (0.0) | 0/14  (0.0) | 0/17 (0.0) | 0/14  (0.0) | 0/12 (0.0) | 0/21  (0.0) | 1/24  (4.2) | 0/16 (0.0) | 1/25  (4.0) | 1/21  (4.8) | |
|  | Vomiting | 0/7 (0.0) | 0/6 (0.0) | 0/8 (0.0) | 0/11 (0.0) | 0/14  (0.0) | 0/17 (0.0) | 1/14  (7.1) | 0/12 (0.0) | 0/21  (0.0) | 0/22  (0.0) | 0/16 (0.0) | 0/25  (0.0) | 0/21  (0.0) | |
|  | Diarrhea | 0/7 (0.0) | 0/6 (0.0) | 0/8 (0.0) | 0/11 (0.0) | 0/14  (0.0) | 0/17 (0.0) | 0/14  (0.0) | 0/12 (0.0) | 0/21  (0.0) | 0/22  (0.0) | 0/16 (0.0) | 0/25  (0.0) | 0/21  (0.0) | |
|  | Itching | 0/8 (0.0) | 0/9 (0.0) | 0/11 (0.0) | 0/15  (0.0) | 0/18  (0.0) | 0/23  (0.0) | 0/17  (0.0) | 0/16  (0.0) | 1/24  (4.2) | 0/24  (0.0) | 0/21  (0.0) | 0/27  (0.0) | 0/24  (0.0) | |
|  | Serious adverse event | 0/7 (0.0) | 0/6 (0.0) | 0/8 (0.0) | 0/11 (0.0) | 0/18  (0.0) | 0/17 (0.0) | 0/14  (0.0) | 0/12 (0.0) | 0/21  (0.0) | 0/22  (0.0) | 0/16 (0.0) | 0/25  (0.0) | 0/21  (0.0) | |
